# *Ehrlichia muris* in *Ixodes cookei* Ticks, Northeastern United States, 2016–2017

**DOI:** 10.3201/eid2406.171755

**Published:** 2018-06

**Authors:** Guang Xu, Patrick Pearson, Stephen M. Rich

**Affiliations:** University of Massachusetts–Amherst, Amherst, Massachusetts, USA

**Keywords:** ticks, Ixodes cookei, Ehrlichia muris, Ixodes scapularis, ticks, bacteria, ehrlichiosis, United States, vector-borne infections

## Abstract

*Ehrlichia muris* is an agent of human ehrlichiosis. To determine its geographic spread in the United States, during 2016–2017, we tested 8,760 ticks from 45 states. A distinct clade of *E. muris* found in 3 *Ixodes cookei* ticks from the northeastern United States suggests transmission by these ticks in this region.

*Ehrlichia muris* was originally isolated from a mouse in Japan in 1983 (*1*). In 2009 in the United States, an *E. muris*–like agent (EMLA) was identified as a causative agent of human ehrlichiosis for 3 symptomatic patients in Wisconsin and 1 in Minnesota (*2*). A retroanalysis of 760 *Ixodes scapularis* ticks collected from 1992 through 1997 in Wisconsin revealed an EMLA infection rate of 0.94%, indicating presence of this pathogen in the upper midwestern region since at least the mid-1990s (*3*). Another study found this infection in 69 patients from 5 states from 2007 through 2013, although all patients had probably been exposed to the ticks in Minnesota or Wisconsin (*4*). In 2017, the *E. muris*–like isolates from the upper midwestern United States were proposed as a taxonomically distinct subspecies, *E. muris* subsp. *eauclairensis* (*5*).

*E. muris* is thought to be transmitted by *Haemaphysalis flava* ticks in Japan, by *I. persulcatus* ticks in eastern Europe, and by *I. ricinus* ticks in western Europe (*5*). Detection of the bacterium in nymphal and adult stages of *I. scapularis* ticks (*2**,**5**,**6*) and in white-footed mice (*Peromyscus leucopus*) suggests that the primary vectors and reservoir hosts of Lyme borreliosis play a major role in the enzootic transmission cycle of *E. muris* in the United States. However, despite the broad distribution of *I. scapularis* ticks and *P. leucopus* mice in North America, to our knowledge, *E. muris* has not been reported outside of Wisconsin and Minnesota (*2**,**7*).

To evaluate the geographic distribution of *E. muris* from May 30, 2016, through October 1, 2017, we used a Taqman real-time PCR to test 8,760 ticks for EMLA, *Anaplasma phagocytophilum*, *Borrelia burgdorferi* sensu lato, *B. miyamotoi*, *B. mayonii, and Babesia microti*. The EMLA test is a modified version of a multiplex Taqman assay and targets the p13 gene (*8*). The human-biting ticks used for this study were submitted to the TickReport public testing program (https://www.tickreport.com) at the University of Massachusetts (Amherst, MA, USA). We confirmed EMLA positivity of the samples by amplifying and sequencing the EMLA citrate synthase (*gltA*) and heat shock protein (*groEL*) genes (*3*). We confirmed the species of EMLA-positive ticks by amplifying and sequencing a partial tick 16S rRNA gene (Technical Appendix). We received 8,760 ticks from 45 states: 243 *Amblyomma americanum*, 2 *A. maculatum*, 7 *Amblyomma* spp., 6 *Dermacentor andersoni*, 3 *D. occidentalis*, 271 *D. variabilis*, 45 *Dermacentor* spp., 14 *I. angustus*, 22 *I. cookei*, 215 *I. pacificus*, 5 *I. ricinus*, 7,800 *I scapularis*, 19 *I. spinipalpis*, 47 *Ixodes* spp., and 7 *Rhipicephalus sanguineus*; 54 ticks were unidentifiable.

We found DNA specific for EMLA in only 2 species of *Ixodes* tick: *I. scapularis* and *I. cookei*. The overall prevalence of EMLA was very low. Only 5 (0.057%) ticks were positive for *E. muris*–specific DNA. Although we tested 7,800 *I. scapularis* ticks from 33 states in the northeastern, midwestern, and southeastern regions, we found only 2 (2/7,800, 0.026%) EMLA-positive *I. scapularis* ticks (1 from Laporte, MN, and 1 from Eleva, WI). These 2 ticks were co-infected with *B. burgdorferi* s. l. and *B. microti*. However, no DNA from *B. miyamotoi*, *B. mayonii*, or *A. phagocytophilum* was detected in these 2 ticks.

The prevalence of EMLA in *I. cookei* ticks was much higher than that in *I. scapularis* ticks. Of the 22 *I. cookei* ticks tested, 3 (3/22, 13.64%) were positive for EMLA (from Holden, ME; Littleton, ME; and Salamanca, NY). Co-infections were not detected in these 3 ticks.

To determine the identity of these EMLA isolates, we examined *gltA* and *groEL* gene sequences of isolates from the 2 *I. scapularis* ticks and found them to be identical. Phylogenetic analysis showed that they clustered with *E. muris* subsp. *eauclairensis*. The *gltA* and *groEL* gene sequences from the 3 *I. cookei* ticks were also identical but formed a new clade between *E. muris* subsp. *eauclairensis* and subsp. *muris* (Figure).

**Figure:**
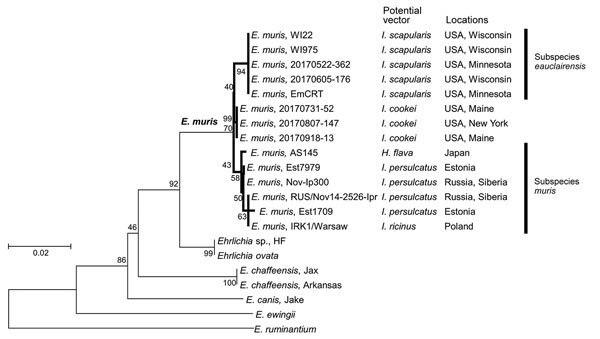
Phylogenetic tree of *Ehrlichia* citrate synthase (*gltA*) and heat shock protein (*groEL*) genes constructed by the maximum-likelihood method of MEGA6 software (http://www.megasoftware.net). The total length of 2 concatenated genes is 1,045 bp. Hasegawa-Kishino-Yano with invariable sites was selected as the best model based on Bayesian information criterion scores. Numbers on the branches represent bootstrap support with 500 bootstrap replicates. Scale bar indicates nucleotide substitutions per site.

The detection of *E. muris* in *I. scapularis* ticks from the upper midwestern United States corroborates previously reported findings (*2**,**3**,**6*). The detection of a distinct clade of *E. muris* in *I. cookei* ticks from the northeastern United States represents a potential risk to humans or a different enzootic cycle of *E. muris* in the Northeast. As primary vectors of Powassan virus (lineage 1), *I. cookei* ticks are widely distributed in eastern North America and are the second most common species of *Ixodes* ticks found on persons in Maine, USA (*9*). Further study is warranted with regard to the vector competence of *I. cookei* ticks for transmitting *E. muris*, the natural enzootic cycle of *E. muris*, and the transmission potential of the pathogen to humans in this region. Meanwhile, human ehrlichiosis should be considered as a possible diagnosis for persons who have been exposed to *I. scapularis* and *I. cookei* ticks in the upper midwestern and northeastern United States, respectively.

Technical Appendix Primers, probes, and DNA sequences used and additional information about ticks positive for *Ehrlichia muris*–like agent in study of *E. muris* in *Ixodes cookei* ticks in the northeastern United States, 2016–2017.
