# Supplementary material for: Ehrlichia muris in Ixodes cookei Ticks, Northeastern United States, 2016–2017
Source: Emerg Infect Dis. 2018 Jun;24(6):1143–4. doi: 10.3201/eid2406.171755 (PMC6004831; doi:10.3201/eid2406.171755)
Supplement: Technical Appendix — Primers, probes, and DNA sequences used and additional information about ticks positive for Ehrlichia muris–like agent in study of E. muris in Ixodes cookei ticks in the northeastern United States, 2016–2017. [file 17-1755-Techapp-s1.pdf]

Article DOI: https://doi.org/10.3201/eid2406.171755

# *Ehrlichia muris* in *Ixodes cookei* Ticks, Northeastern United States, 2016–2017

## Technical Appendix

**Technical Appendix Table 1**. Primers and probes used in this study

| Target gene | Application | Type | Sequences (5′→3′) | Tm (C) | Reference |
|---|---|---|---|---|---|
| 16S | Tick species PCR and confirmation | Forward | TGCTGTAGTATTTTGACTATACAAAGG | 55 | This paper |
| | | Reverse | ATCCTAATCCAACATCGAGGTC | | |
| ITS | *Ixodes scapularis* identification | Forward | TGCGTTTTCTTTGAGCAAATGCACGAG | 60 | This paper |
| | | Reverse | GTACGGGATTTTCCACAAACGGTATCCA | | |
| | | Probe | FAM-TGCGCTTAACCAGTCCTCCTCCTCCTACGA-BHQ | | |
| ITS | *Ixodes pacificus* identification | Forward | CTCGGAGCAAGTACGGAGGTAG | 60 | This paper |
| | | Reverse | TTTCCACAAAACGGTCGCCATC | | |
| | | Probe | Cy5-CTGAGCCAAGTCCTCTTCCTACCCGGTTTG-BHQ | | |
| ITS | *Amblyomma americanum* identification | Forward | CGACCGCCGCAGGAAGG | 60 | This paper |
| | | Reverse | CGTTTCTCGCAGCAGTTCGG | | |
| | | Probe | FAM-CCCGCTGGCCCGCGTACGTGT-BHQ | | |
| ITS | *Dermacentor variabilis* identification | Forward | CTGAAGATTCTTTGCGAGGAGCGG | 60 | (*1*) |
| | | Reverse | GCGTCAGCTCGGCCAAC | | |
| | | Probe | FAM-AGAAGGGCGTGCCCCGAAAGCGG-BHQ | | |
| gltA | *E. muris* PCR and confirmation | Forward | TACAGATTTCTCAAGAATATACA (outer)<br>TGGCATGTTTTTCTGCCTTA (inner) | 50 | (*2*) |
| | | Reverse | AATGCAATGTTTTCTAATTCTAC (outer)<br>TGACCAAAACCCATTAATCTTG (inner) | | |
| groEL | *E. muris* PCR and confirmation | Forward | GGATCCATTGGCTCTTGCTA (outer)<br>AAGGGATTCAAAGAATTGGATG (inner) | 50 | (*2*) |
| | | Reverse | CCACCAACCTTTAAGACAGCA (outer)<br>CCACCAACCTTTAAGACAGCA (inner) | | |
| P13 | EMLA detection | Forward | TACCTAATTCTTCTCAAGAGATTCAGTTG | 60 | This paper |
| | | Reverse | ATGATGATACTGCGAACAACTATAAGAG | | |
| | | Probe | Cy5-ATATTGATAAAAGAGTCAGTGTTGATCCGTATGAGTTAGGGTT-BHQ | | |
| glpQ | *Borrelia miyamotoi* detection | Forward | GACATAGTTCTAACAAAGGACAATATTCC | 60 | (*3*) |
| | | Reverse | TCCGTTTTCTCTAGCTCGATTGG | | |
| | | Probe | HEX-TGCACGACCCAGAAATTGACACAACCACAA-BHQ | | |
| ospA | *Borrelia burgdorferi Sensu Lato* detection | Forward | ATAGGTCTAATATTAGCCTTAATAGCAT | 60 | This paper |
| | | Reverse | AGATCGTACTTGCCGTCTT | | |
| | | Probe | FAM-aagc+Aaa+Atgtt+Agc+Agccttga-BHQ (LNA) | | |
| Tubulin | *Babesia* detection | Forward | GATTTGGAACCTGGCACCATG | 60 | (*4*) |
| | | Reverse | AATGACCCTTAGCCCAATTATTTCC | | |
| | | Probe | FAM-ATCTGGCCCATACGGTGAATTGTTTCGC-BHQ | | |
| MSP-2 | *Anaplasma* detection | Forward | ATGGAAGGTAGTGTTGGTTATGGTATT | 60 | (*4*) |
| | | Reverse | TTGGTCTTGAAGCGCTCGTA | | |
| | | Probe | HEX-TGGTGCCAGGGTTGAGCTTGAGATTG-BHQ | | |

*We use species-specific taqman qPCR identified *Amblyomma americanum* (243), *Dermacentor variabilis* (271), *Ixodes pacificus* (215), and *Ixodes scapularis* (7800). A fragment of 16S mtDNA was used to identify EMLA-positive *Ixodes scapularis* ticks (2), *Ixodes angustus* (14), *Ixodes cookei* (22), *Ixodes ricinus* (5), *Ixodes spinipalpis* (19) and *Dermacentor occidentalis* (3). The rest 114 ticks were identified by morphological characters or marked as unidentifiable because of poor sample conditions.

**Technical Appendix Table 2.** DNA sequences used in this study

| Organism | Gene | Sample or strain | GenBank accession no.* |
|---|---|---|---|
| *Ixodes scapularis* | 16s rRNA | 20170522–362 | MG242324 |
| *Ixodes scapularis* | 16s rRNA | 20170605–176 | MG242325 |
| *Ixodes cookei* | 16s rRNA | 20170731–52 | MG242326 |
| *Ixodes cookei* | 16s rRNA | 20170807–147 | MG242327 |
| *Ixodes cookei* | 16s rRNA | 20170918–13 | MG242328 |
| *E. muris* | gltA | 20170522–362 | MG242314 |
| *E. muris* | gltA | 20170605–176 | MG242315 |
| *E. muris* | gltA | 20170731–52 | MG242316 |
| *E. muris* | gltA | 20170807–147 | MG242317 |
| *E. muris* | gltA | 20170918–13 | MG242318 |
| *E. muris* | groEL | 20170522–362 | MG242319 |
| *E. muris* | groEL | 20170605–176 | MG242320 |
| *E. muris* | groEL | 20170731–52 | MG242321 |
| *E. muris* | groEL | 20170807–147 | MG242322 |
| *E. muris* | groEL | 20170918–13 | MG242323 |
| *E. muris* | gltA | WI22 | HQ660494 |
| *E. muris* | groEL | WI22 | HQ660492 |
| *E. muris* | gltA | WI975 | HQ660497 |
| *E. muris* | groEL | WI975 | HQ660493 |
| *E. muris* | gltA | EmCRT | LANU00000000 |
| *E. muris* | groEL | EmCRT | LANU00000000 |
| *E. muris* | gltA | AS145 | CP006917 |
| *E. muris* | groEL | AS145 | CP006917 |
| *E. muris* | groEL | Est7979 | KU535864 |
| *E. muris* | groEL | Nov-Ip300 | GU358687 |
| *E. muris* | groEL | RUS/Nov14–2526-Ipr | KX980049 |
| *E. muris* | groEL | Est1709 | KU535861 |
| *E. muris* | groEL | IRK1/Warsaw | KF312362 |
| *Ehrlichia spp.* | gltA | HF | NZ_CP007474 |
| *Ehrlichia spp.* | groEL | HF | NZ_CP007474 |
| *E. ovata* | groEL | Shizuoka | DQ672553 |
| *E. chaffeensis* | gltA | Jax | CP007475 |
| *E. chaffeensis* | groEL | Jax | CP007475 |
| *E. chaffeensis* | gltA | Arkansas | CP000236 |
| *E. chaffeensis* | groEL | Arkansas | CP000236 |
| *E. canis* | gltA | Jake | NC_007354 |
| *E. canis* | groEL | Jake | NC_007354 |
| *E. ewingii* | gltA | Panola Mountain | DQ365879 |
| *E. ewingii* | groEL | | AF195273 |
| *E. ruminantium* | gltA | Welgevonden | NC_005295 |
| *E. ruminantium* | groEL | Welgevonden | NC_005295 |

*GenBank accession numbers MG242314 to MG242328 are new sequences in this study.

**Technical Appendix Table 3**. EMLA positive ticks in this study

| Tick ID# | Tick species | Tick stage | Source | Location |
|---|---|---|---|---|
| 20170522–362 | *Ixodes scapularis* | Adult | Human | Laporte, MN 56461 |
| 20170605–176 | *Ixodes scapularis* | Adult | Human | Eleva, WI 54738 |
| 20170731–52 | *Ixodes cookei* | Adult | Human | Holden, ME 04429 |
| 20170807–147 | *Ixodes cookei* | Adult | Human | Salamanca, NY 14779 |
| 20170918–13 | *Ixodes cookei* | Adult | Human | Littleton, ME 04730 |

**References**


1. Shone SM, Dillon HJ, Hom SS, Delgado N. A novel real-time PCR assay for the speciation of medically important ticks. Vector Borne Zoonotic Dis. 2006;6:152–60. PubMed http://dx.doi.org/10.1089/vbz.2006.6.152

2. Telford Iii SR, Goethert HK, Cunningham JA. Prevalence of *Ehrlichia muris* in Wisconsin Deer Ticks Collected During the Mid 1990s. Open Microbiol J. 2011;5:18–20. PubMed http://dx.doi.org/10.2174/1874285801105010018

3. Krause PJ, Schwab J, Narasimhan S, Brancato J, Xu G, Rich SM. Hard Tick Relapsing Fever Caused by Borrelia miyamotoi in a Child. Pediatr Infect Dis J. 2016;35:1352–4. PubMed http://dx.doi.org/10.1097/INF.0000000000001330

4. Xu G, Mather TN, Hollingsworth CS, Rich SM. Passive Surveillance of *Ixodes scapularis* (Say), Their Biting Activity, and Associated Pathogens in Massachusetts. Vector Borne Zoonotic Dis. 2016;16:520–7. PubMed http://dx.doi.org/10.1089/vbz.2015.1912
